# Voltage sensing mechanism in skeletal muscle excitation-contraction coupling: coming of age or midlife crisis?

**DOI:** 10.1186/s13395-018-0167-9

**Published:** 2018-07-19

**Authors:** Erick O. Hernández-Ochoa, Martin F. Schneider

**Affiliations:** Department of Biochemistry and Molecular Biology, University of Maryland School of Medicine, 108 N. Greene Street, Baltimore, MD 21201 USA

**Keywords:** Skeletal muscle, Excitation-contraction coupling, Charge movement, Voltage sensors, DHPR/Cav1.1, L-type voltage-gated calcium channel, Ca^2+^ release, RyR1

## Abstract

The process by which muscle fiber electrical depolarization is linked to activation of muscle contraction is known as excitation-contraction coupling (ECC). Our understanding of ECC has increased enormously since the early scientific descriptions of the phenomenon of electrical activation of muscle contraction by Galvani that date back to the end of the eighteenth century. Major advances in electrical and optical measurements, including muscle fiber voltage clamp to reveal membrane electrical properties, in conjunction with the development of electron microscopy to unveil structural details provided an elegant view of ECC in skeletal muscle during the last century. This surge of knowledge on structural and biophysical aspects of the skeletal muscle was followed by breakthroughs in biochemistry and molecular biology, which allowed for the isolation, purification, and DNA sequencing of the muscle fiber membrane calcium channel/transverse tubule (TT) membrane voltage sensor (Cav1.1) for ECC and of the muscle ryanodine receptor/sarcoplasmic reticulum Ca^2+^ release channel (RyR1), two essential players of ECC in skeletal muscle. In regard to the process of voltage sensing for controlling calcium release, numerous studies support the concept that the TT Cav1.1 channel is the voltage sensor for ECC, as well as also being a Ca^2+^ channel in the TT membrane. In this review, we present early and recent findings that support and define the role of Cav1.1 as a voltage sensor for ECC.

## Background

In skeletal muscle, electrical impulses carried by the axons of motoneurons travel to the nerve endings at the muscle endplate (the muscle synapse), where these electrical signals are converted into chemical signals that produce depolarizing postsynaptic potentials at the neuromuscular junction sarcolemma of the muscle fiber [1, 2]. In all but a few “tonic” muscle fibers, these postsynaptic endplate potentials elicit a further depolarization of the muscle fiber, carried out by skeletal muscle voltage-gated sodium channels, initiating and propagating the muscle action potential [3–5]. The muscle action potential (AP) travels both longitudinally away from the fiber endplate along the muscle fiber surface sarcolemma and radially into the fiber via invaginations of the sarcolemma that form the transverse tubular (TT) system [6, 7]. The AP depolarization activates skeletal muscle voltage-gated calcium channels (Cav1.1; also known as dihydropyridine receptors, DHPR) [8]. The Cav1.1 channels serve as the voltage sensing machinery for the process of TT depolarization-induced calcium release from the sarcoplasmic reticulum [9] via intracellular sarcoplasmic reticulum (SR) calcium release channels, the type 1 ryanodine receptors (RyR1) [10]. This process that begins with the muscle AP propagation and results in muscle contraction is known as excitation-contraction coupling (ECC). This term was coined by Sandow in the 1950s [11] to include these main events critical for muscle activation, well before the molecular identities or even the existence of the molecular players (Cav1.1 and RyR1) was identified or established. Since then we have accumulated an incredible amount of information concerning the structural aspects and molecular and functional details of the ECC process.

The knowledge that the muscle contraction was controlled by electrical signals was already established by the pioneering work of Galvani, Volta, and Walsh [12, 13]. Subsequent studies and discoveries by Nobili, Matteucci, Du Bois-Reymond, and Ringer, to mention just a few of the pillars, formed the foundations for modern understanding of bioexcitability of muscle and other excitable tissues [14]. The remarkable work by Hodgkin, Huxley, and Katz [15–19] established the playing field for the subsequent wave of functional studies dealing with excitability in general, and with the TT voltage sensor for EEC in particular, as we consider here.

## Introduction to excitation-contraction coupling

By the 1950s and 1960s, the processes that initiated and accompanied skeletal muscle contraction had been studied from several angles [20, 21]. Muscle biologists and physiologists were working collectively trying to decipher the details of the machinery that controls the process of muscle contraction using state-of-the-art techniques from that period. These pioneers made important fundamental contributions to understanding ECC, including the following. (1) Hodgkin and Horowicz [22, 23] proposed that the event that normally induces muscle contraction is a change in membrane potential rather than the longitudinal spread of current along the fiber; they also showed that the development of tension was dependent on membrane potential and was described by a steep sigmoidal curve of tension as a function of membrane potential. (2) The experiments of Huxley and Taylor [7] showing activation at the Z disk in frog muscle fibers and of Huxley and Straub showing local activation at the A-I band junction in lizard muscle [24], together with the localization of the TT system at the Z disk in frog muscle [25] and at the A and I band junction in lizard muscle [26], indicated that the transverse tubules (TT) of the skeletal muscle fibers form the network which conducts the surface depolarization radially into a muscle fiber to initiate contraction. (3) Investigations started by Ringer [27] and continued by Heilbrunn [28], Kamada and Kinoshita [29], and others introduced the role of Ca^2+^ as key regulator of striated muscle activation. Further details of the complex action of Ca^2+^ on muscle contractile activation were eventually provided by Weber [30, 31] and Ebashi [32]; reviewed in more detail by Endo [33]. (4) Robertson [26], Andersson-Cedegren [34], Francini-Armstrong and Porter [35], and Peachey [36], using electron microscopy, described that the ultrastructure of transverse tubules (TT) and that the terminal cisternae of the SR are in close proximity to the TTs.

This year (2018) is the 45th anniversary of the demonstration of ECC voltage sensor charge movement [37]. In keeping with the theme of “coming of age/midlife crisis” of the ECC voltage sensor, here we will first review the discovery and early functional studies of the ECC voltage sensor and its role and properties, largely carried out during the last quarter of the twentieth century. We then consider more recent molecular, structural, and mechanistic studies, as well as possible future directions. A detailed review of the cloning of the Cav1.1 and RyR, and identification of their skeletal muscle isoforms as the ECC voltage sensor and skeletal muscle SR Ca^2+^ release channel, respectively, is beyond the present scope and can be found elsewhere [38–42].

## Intramembrane charge movement and ECC

### Voltage sensor charge movements were predicted by Hodgkin and Huxley

**Fig. 1:**
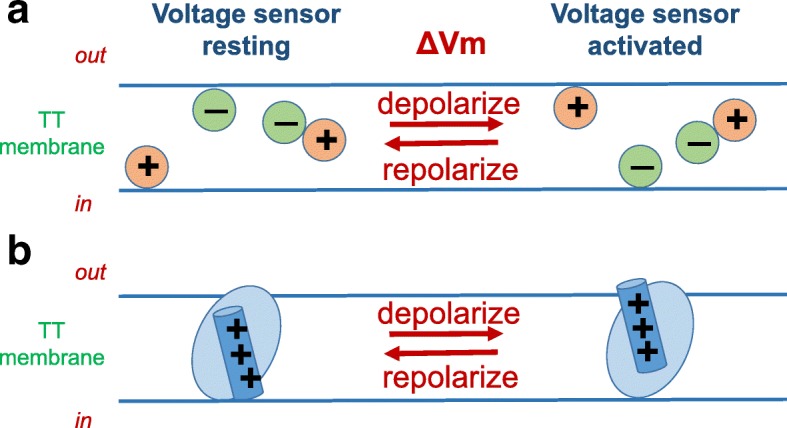
Hypothetical mechanisms for a mobile charged intramembrane voltage sensor. **a** Voltage-dependent intramembrane charge movement could be generated by positive charges moving outward during depolarization, negative charges moving inward, or dipoles rotating. In each, the charge would return to its starting distribution on membrane repolarization, so the charge moved outward during membrane depolarization must equal the charge moving back on repolarization. **b** Cartoon illustration of current concept of intramembrane voltage sensors as positively charged amino acid residues on transmembrane alpha helices. As in **a**, the charge moved outward during depolarization (here on an alpha helix) will equal the charge returning on repolarization

In their classic work on the membrane potential-dependent ionic conductances underlying the nerve axon action potential, Hodgkin and Huxley [15] predicted that any voltage-sensitive process, such as voltage-dependent Na^+^ or K^+^ conductance, should be controlled by mobile charges that are trapped within the membrane but can be displaced in response to changes in electrical potential energy due to changes in transmembrane voltage. They further predicted that such intramembrane charges should give rise to tiny charge displacement currents in response to changes in transmembrane voltage (Vm), as the putative charges trapped within the membrane redistribute within the membrane in response to the change in electrical potential. However, charge displacement currents were not detected by Hodgkin and Huxley [15]. In fact, it took over two decades to prove the charge movement hypothesis of Hodgkin and Huxley [15, 37]. In addition, at the time of Hodgkin and Huxley, and continuing well through the time of the early experimental studies characterizing the functional properties of voltage sensor charge movements, the molecular identity of the voltage sensors was not even known. Consequently, it was unknown at the time whether the putative and subsequently measured intramembrane charge movements were generated by (1) positive charges held near the inside of the membrane at the inside-negative resting potential, moving outward during depolarization (Fig. 1a, right) and returning inward after repolarization (Fig. 1a, left); (2) negative charges positioned near the outside of the membrane at rest, moving inward during membrane depolarization and returning outward during repolarization (Fig. 1a, left); or even (3) dipolar charges rotating in the membrane as the positive end moves outward and the negative charge moves inward during depolarization and reverses this movement during repolarization (Fig. 1b). Subsequently, with the establishment of the molecular identity, amino acid sequence, and predicted or experimentally determined molecular structure of the ECC voltage sensor (Cav1.1) [38, 43], as well as those of other voltage-sensitive channels, it is now accepted that positively charged transmembrane alpha helical “S4” segments in membrane-spanning domains (Fig. 1b; considered in detail below) are the electrically charged molecular components that serve as voltage sensors for both ECC and channel gating of plasma membrane and TT Na^+^, Ca^2+^, and K^+^ channels [44].

### Voltage sensor charge movements were first detected in skeletal muscle fibers

The first successful intentional measurement of the voltage sensor charge displacement currents predicted by Hodgkin and Huxley was carried out on skeletal muscle fibers. Assuming that the charge displacement currents would be small, Schneider and Chandler [37] voltage clamped frog skeletal muscle fibers in the presence of blockers for each of the major ionic conductances (TTX for Na^+^ conductance, Rb^+^ replacing K^+^ for K^+^ conductance, methanesulphonate for Cl− conductance). Under these conditions, ionic currents were essentially absent. However, the linear capacitative current, needed to charge the linear capacitance of the muscle fiber lipid bilayer membrane when the fiber membrane potential was changed, still remained and obscured the putative voltage sensor charge movement.

**Fig. 2:**
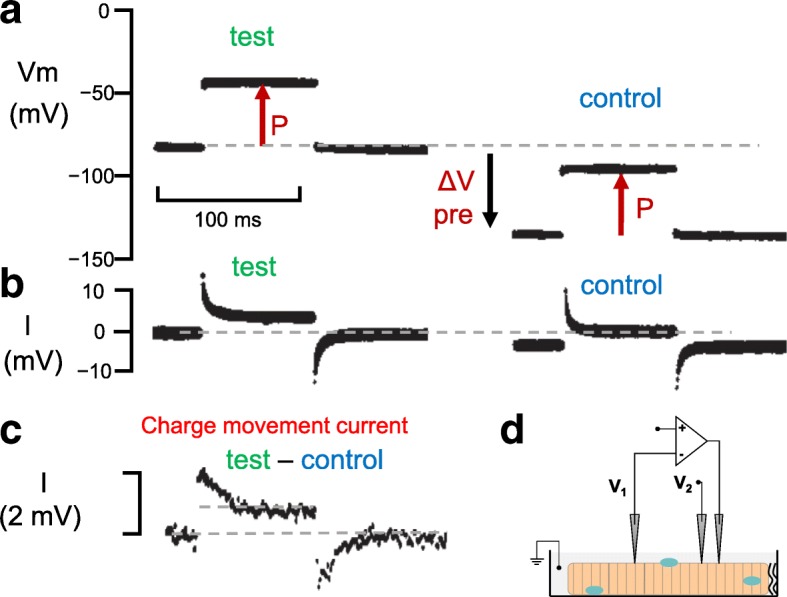
Protocol for original recording of intramembrane charge movements. **a** Pulse protocol used to extract current carried by intramembrane charge movements. The same pulse (P) was applied either from the holding potential (test, left) or superimposed on a hyperpolarizing prepulse (∆ Vpre; control, right). The pulse over the control voltage range is assumed to cause no intramembrane charge movement. **b** The total current recordings for the pulses in **a**. Traces in **a** and **b** were recorded as photos of oscilloscope display. **c** Difference between membrane current in the test pulse minus the current in the control pulse, obtained by digital subtraction of digital recordings of test and control currents using an analog to digital converter and a digital recording system. **d** Schematic diagram of the three microelectrode voltage clamp systems used in this experiment. All records obtained at the tendon termination of a muscle fiber in a frog sartorius muscle stretched to reduce contraction and bathed in solution to block or remove essentially all membrane conductance. From ref. [37], with modification

A strategy was needed for removing the linear capacitative current in order to “unmask” the current carried by voltage sensor charge movement. The expected voltage sensor charge displacement current was anticipated to saturate at highly positive or highly negative membrane potentials, as all mobile charges were maximally displaced during large depolarizations or hyperpolarizations (Fig. 1). Furthermore, the voltage sensor charge displacements were expected to occur over the Vm range where muscle fiber contraction was activated, roughly between about − 50 and + 20 mV [23]. Thus, the putative muscle voltage sensor charge displacement current was predicted to be an “extra” non-linear component of the total membrane current. Formally, this extra current was “capacitative” in nature since whatever charge moved outward during depolarization was trapped within the membrane and was obliged to move back to its starting intramembrane location when the membrane was repolarized (Fig. 1). To extract the non-linear component from the total measured membrane capacitative current for each “test” pulse (P) applied from the holding potential (Fig. 2a, left), the same depolarizing pulse (P) was superimposed on a negative prepulse (ΔV pre) of larger absolute amplitude than the test pulse (Fig. 2a, right) [37]. In this way the same amplitude pulse (P) was now applied over a Vm range that was entirely negative to the initial holding potential. This pulse served as the “control” pulse and is assumed to contain only the linear capacitative current (Fig. 2b, right).

The currents for both the test and control pulses were recorded digitally during the experiment using a digital signal averaging device, which preceded the introduction of laboratory computers and the use of PCs. Using the digitized records of total current, the current recorded for the “control” depolarization (in the range negative to the holding potential; Fig. 2b, right) was digitally subtracted from the current for the “test” depolarization (over a voltage range positive to the holding potential; Fig. 2b, left) to give the “non-linear” membrane current (Fig. 2c). Assuming any remaining non-linear ionic current to be constant (i.e., time-independent) during the pulse, the time-dependent component of the non-linear current (test−control) was taken to be the non-linear “charge displacement current” or “charge movement current” due to the voltage sensor movement. This current is the current in excess of the steady non-linear current during the pulse (Fig. 2c; upper dashed line during the pulse), and the current due to the return of the voltage sensors was taken to be the current below the initial and final zero current (Fig. 2c; dashed line after the pulse) when the voltage sensors return to their starting distribution after the pulse. These initial recordings of voltage sensor currents were made at the end of a single muscle fiber in an isolated frog sartorius muscle using the three microelectrode voltage clamp system developed by Adrian et al. (Fig. 2d; [45, 46]).

### Critical steps: voltage sensor currents and charge movements in skeletal muscle

**Fig. 3:**
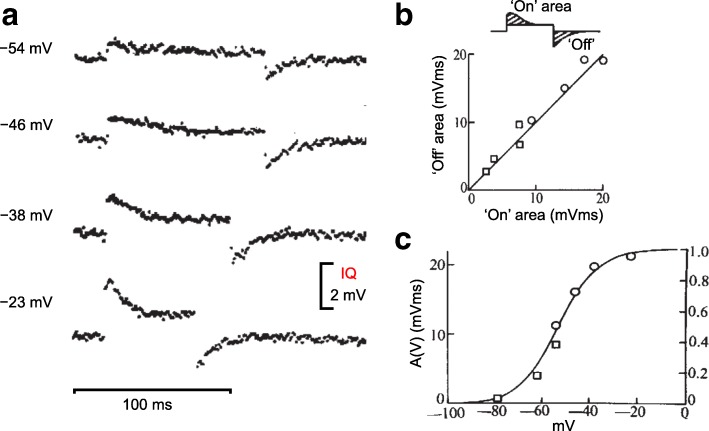
Initial characterization of intramembrane charge movement currents. **a** Non-linear difference currents between test pulses to the indicated membrane potentials and corresponding control pulses covering a membrane potential range negative to the holding potential. Same protocol as in Fig. 1. Pulse duration was decreased for the two largest depolarizations displayed. **b** Equality of charge moved during the “on” and “off” of different amplitude test pulse depolarizations. **c** Voltage dependence of non-linear charge moved for test pulses to the indicated membrane potentials. All records and graphs from ref. [37], with modification

The original voltage sensor charge movement currents detected during and after pulses to a range of membrane potentials using the pulse protocol in Fig. 2a are shown in Fig. 3a [37]. The voltage sensor current amplitude both during and after the pulses increased with increasing depolarization (Fig. 3a). The time course of the charge movement currents during the pulse became increasingly rapid as the depolarizing pulses were increased in amplitude. In contrast, the time course of the charge movement current after the pulse (i.e., during fiber repolarization to the initial holding potential) did not noticeably change in kinetics as the pulse depolarization was increased (Fig. 3a). As discussed further below, from the original report, the charge movement kinetics and voltage dependence were generally in the range that would be appropriate for muscle contractile activation, so it was not unreasonable to identify these charge movements with the intramembrane movement of ECC voltage sensors in the TT membrane [37]. With subsequent sophistication of experimental and recording procedures, records with better signal to noise and corresponding resolution of kinetic details were obtained (a) from frog individual muscle fibers in a single Vaseline gap voltage clamp system which allowed fiber movement without movement artifacts in the membrane current records during contractile activation [47, 48], (b) from frog fibers studied in a double Vaseline gap when stretched to eliminate mechanical movement and the corresponding movement artifacts during activation [49, 50], and (c) from whole cell voltage clamped mammalian short skeletal muscle fibers adhering to a glass coverslip [51–54].

As embodied in the cartoons in Fig. 1, any hypothetical positive voltage sensor charges that moved outward within the membrane during fiber depolarization (or any hypothetical negative voltage sensor charges that moved inward) are expected to return to their initial intramembrane location when the fiber was repolarized to its initial membrane potential. Thus, the amount of voltage sensor charge moved outward during depolarization was anticipated to equal the amount of voltage sensor charge moved inward during the repolarization. This “on/off” equality of amounts of charge moving outward during depolarization and inward during repolarization was the expected signature of voltage sensor charge displacement currents (Fig. 3b). From the first report of measurements of the ECC voltage sensor currents in muscle fibers, it was established that the on/off equality criterion was in fact fulfilled both for the charge movement currents generated by test pulses to various membrane potentials (Fig. 3b) as well as by pulses of various durations to the same membrane potential [37].

### Voltage dependence of charge movement, and its first interpretation

The measured amount of charge moved increased in a sigmoidal manner as a function of increasing membrane depolarization from the resting holding potential and approached saturation for the largest depolarizations used (Fig. 3c). The voltage dependence of charge moved was interpreted using a model in which a single uniform population of intramembrane charges were each assumed to occupy one of two possible membrane locations that differed in energy by a fraction of the full electrical potential energy across the membrane. Non-linear charge movement (*Q*) had a sigmoidal dependence on test membrane potential, according to a two-state Boltzmann function:

$$ Q=Q\max /\left[1+\exp\ \left(\left(-V+ Vh\right)/k\right)\right] $$

where *Qmax* is the maximum charge (per unit of linear capacitance), *Vh* is the mid-point, and *k* a measure of steepness (Fig. 3c, continuous line through symbols). While this procedure allows for an approximation of the voltage dependence of the charge movement in ECC, it may not be adequate to estimate total charge (i.e., total number of elementary charges), especially if the charge moves in multiple sequential steps [55].

### Voltage sensor charge moved predicts pulse durations needed to give detectable contraction

**Fig. 4:**
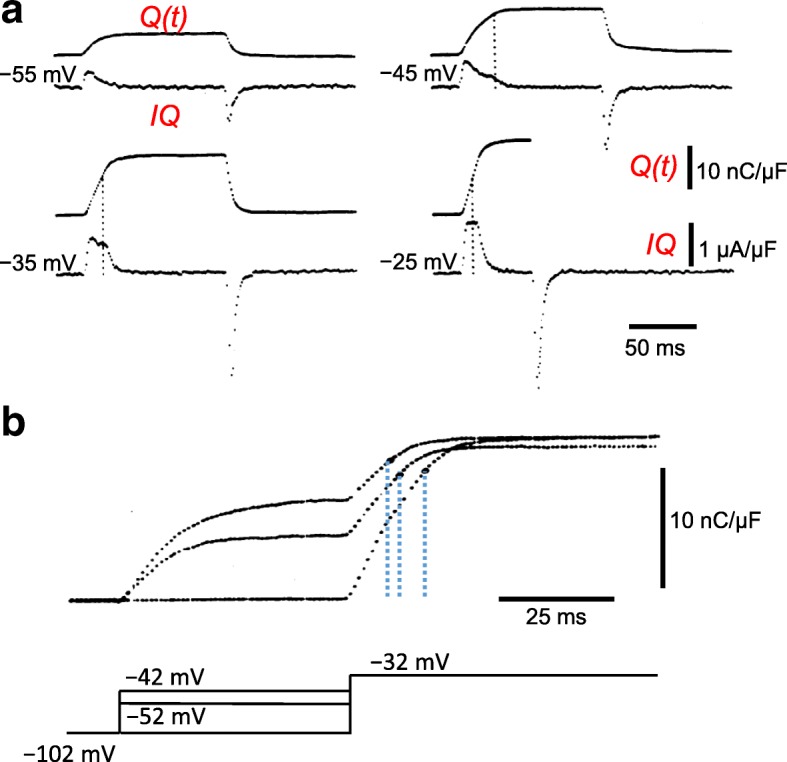
Depolarizing pulses that produce a just-detectable muscle fiber movement displace a set (“threshold”) amount of charge, which can be termed “pre-activating” charge since it must move in order to attain detectable fiber activation. **a** Charge movement records during muscle fiber depolarization to indicated voltages. The dashed vertical lines indicate the pulse duration needed to give a microscopically just-detectable fiber movement for shorter pulses to the same voltage. No contraction was detected in at − 55 mV. **b** Pulse to − 32 mV applied alone or together with indicated prepulses, which move only pre-activating charge, since no contraction was detected during the prepulses alone. Charges moved for just-detectable fiber movement (height of black dots) in the test pulses were the same with or without prepulses at the pulse durations for just-detectable fiber contraction (dashed vertical lines). The prepulses decrease the pulse duration required to reach detectable fiber contraction during the test pulse, and this decrease was equal to the time to move the prepulse (pre-activating) charge at the test pulse voltage. Reproduced, with modification from ref. [47]

An immediate question that arose after the first detection of charge movement currents was whether the voltage sensor currents detected in muscle fibers were in fact the control system for depolarization-induced contractile activation. Two early studies addressed this question, using different pulse protocols to show that voltage sensor charge movement measurements can be used to closely predict the initiation of muscle contraction. First, it was previously well-established that during prolonged (10s of sec) fiber depolarization, fibers first contracted and then became mechanically relaxed [23]. During similar prolonged voltage clamp depolarizations, muscle voltage sensor charge displacement properties were also modified [56]. Comparing the time course of recovery of charge movement after repolarization of fully depolarized fibers with the time for recovery of just-detectable contraction during repolarization of a depolarized fiber, it was found that charge recovery could predict the recovery of contractile ability, implying a close relationship between charge movement and contractile activation [57]. Second, during voltage clamp depolarization of fully polarized fibers, the pulse duration required to produce a microscopically just-detectable contraction at different depolarizations moved a constant amount of voltage sensor charge [47, 48]. In this experiment, non-linear capacitive currents (*IQ*(*t*)), charge movement (*Q*(*t*)), and the occurrence of just-detectable contraction were all monitored in the same single muscle fiber (Fig. 4a). Contraction was elicited by test pulses to − 45, − 35, and − 25 mV, but not by the smallest test pulse to − 55 mV (Fig. 4a). Furthermore, using a test pulse alone (to − 32 mV) or together with two different amplitude prepulses (Fig. 4b, bottom), which alone did not produce detectable contraction, it was found that the prepulses decreased the time needed to reach contractile threshold during the test pulse (Fig. 4b). The shortening of the test pulse duration for just-detectable contraction could be predicted from the charge movement recordings as the time to move the prepulse charge at the test pulse voltage. These studies demonstrated a close correlation between the voltage sensor charge movement and just-detectable contractile activation of muscle fibers. The charge required to attain a just-detectable contraction is here termed “pre-activating” charge since it must be moved in a step or sequence of steps prior to the step(s) that actually activate contraction, but it does not itself activate contraction (discussed further below).

### Voltage sensors control other membrane potential-dependent processes

As predicted by Hodgkin and Huxley [15], any Vm-sensitive process was expected to involve a voltage sensor charge movement. Indeed, shortly after the initial measurements of intramembrane charge movement in skeletal muscle fibers, analogous charge displacement currents were monitored in squid axons [58]. Based on their properties, the intramembrane charge movements detected in axons were identified as “gating currents” for the axon Na^+^ channels. Following the initial work of Schneider and Chandler in muscle and of Armstrong and Bezanilla in axons, charge movement of voltage sensors has been used extensively to study channel gating kinetics and putative voltage-dependent molecular rearrangements in a variety of voltage-sensitive channels [59–62] and even in membrane potential-dependent enzymes, pumps, and receptors [63–65]. Over the years, various pulse protocols (“P/n”, +/− P) have been devised to extract the non-linear capacitative current (the “charge movement current” or “gating current”) from the total capacitative current [44, 66, 67], in addition to the P test–P control protocol (Fig. 2a, b) developed for the initial measurements of muscle voltage sensor charge movement [37].

### A multi-tasking Ca^2+^ channel: the ECC voltage sensor controls two distinct Ca^2+^ channels in two different membranes

**Fig. 5:**
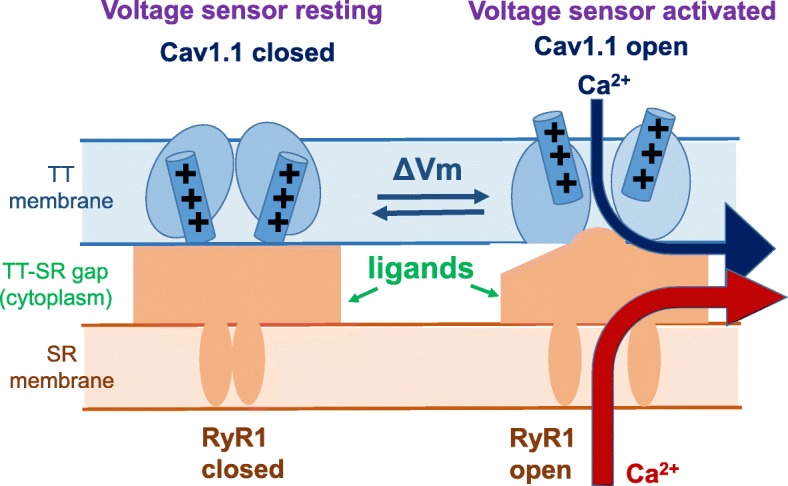
Cav1.1 (pale blue) serves as voltage sensor for two different Ca^2+^ channels: its own intramolecular Ca^2+^ channel in the TT membrane (current illustrated in blue) and the RyR1 Ca^2+^ release channel (tan) in the SR membrane (current illustrated in red). Cartoon representation of the simplest gating mechanism. RyR1 Ca^2+^ channel is directly controlled by molecular coupling of Cav1.1 to RyR1. Note that Ca^2+^ influx via the TT Cav1.1 Ca^2+^ channel is not needed for activation of the RyR1 SR Ca^2+^ release channel

Activation of the TT voltage sensor within the Cav1.1 molecule controls two different TT voltage-sensitive Ca^2+^ channels [68]. First, Cav1.1 voltage sensor movement leads to opening of the ion conducting Ca^2+^ channel within the Cav1.1/ECC voltage sensor molecule itself [69, 70]. This allows Ca^2+^ influx across the TT membrane and into the cytoplasm (blue curved arrow in Fig. 5), which is manifested as L-type inward Ca^2+^ current across the voltage clamped TT system. Second, the Cav1.1 voltage sensor movement promotes opening of the SR RyR1/Ca^2+^ release channel [8, 9], allowing Ca^2+^ release from the SR (red arrow in Fig. 5; discussed in detail below). It is crucial to note that even though isolated RyR1/Ca^2+^ release channels of skeletal muscle can be activated by elevated Ca^2+^ [71, 72], it is well established that Ca^2+^ entry via the Cav1.1 Ca^2+^ channel current is *not* required for activation of RyR1 Ca^2+^ release during muscle fiber depolarization [73], where depolarization beyond the reversal potential for L-type Ca^2+^ current [74] or in zero Ca^2+^ external with EGTA [75], which eliminates inward Ca^2+^ current, does *not* alter muscle activation. Indeed, “skeletal” type of ECC is defined as being Ca^2+^ influx-independent ([76]; see further discussion below).

### Monitoring and characterizing TT membrane depolarization-induced SR Ca^2+^ release

An important experimental distinction exists between the two Ca^2+^ channels regulated by the TT Cav1.1 voltage sensor. L-type Ca^2+^ current can be monitored directly using the same voltage clamp circuit as used for monitoring voltage sensor charge movement [69, 70]. In contrast, SR Ca^2+^ release occurs across the SR membrane, which is *not* part of the electrical circuit for current flow between the cytoplasm and bathing solution that is monitored by the voltage clamp circuit. Consequently, SR Ca^2+^ release cannot be monitored by the voltage clamp system. A second experimental measuring system and analysis procedure is needed to calculate SR Ca^2+^ release.

**Fig. 6:**
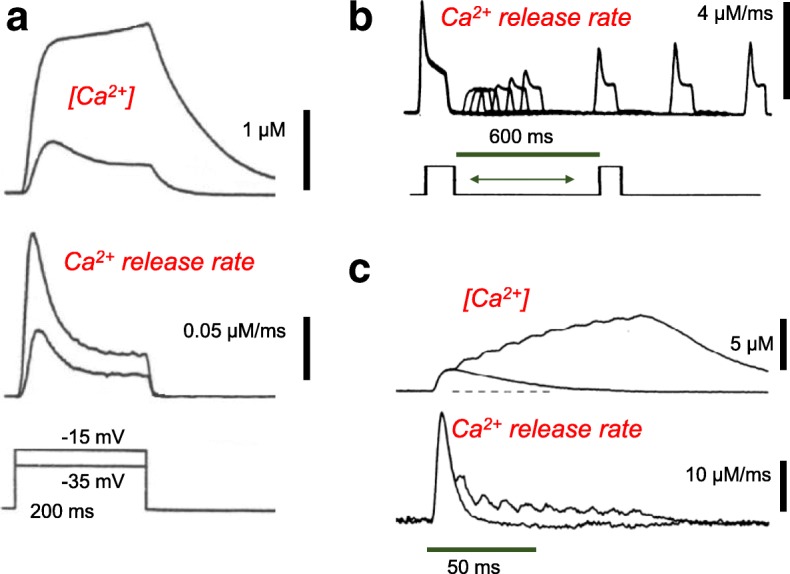
Rate of Ca^2+^ release from the SR during muscle fiber depolarization calculated from the myoplasmic Ca^2+^ transients measured experimentally in individual muscle fibers. **a** Measured Ca^2+^ transients (top) and corresponding calculated time course of rate of Ca^2+^ release from the SR (middle) for voltage clamp depolarizations to indicated membrane potentials (bottom). The rate of Ca^2+^ release reaches an early peak and then declines appreciably during continued depolarization. Reproduced, with modification from ref. [78]. **b** Time course of recovery of Ca^2+^ release following an initial inactivating pulse, followed at various times by a repeat application of the same pulse. After a lag of about 100 ms, the early peak begins to recover and is fully recovered by 600 ms. However, at 600 ms recovery, the release wave form is still smaller than in the initial pulse and recovers much more slowly, indicating recovery from a second process, which was attributed to recovery from SR Ca^2+^ depletion. Reproduced, with modification from ref. [83]. **c** Ca^2+^ transients (top) and rate of SR Ca^2+^ release (bottom) calculated from the measured Ca^2+^ time courses for a single action potential or for a train of action potentials. Release in the second and later action potentials is considerably reduced compared to the release in the first action potential. Reproduced, with modification from ref. [79]

The first step in determining SR Ca^2+^ release is to monitor the free myoplasmic Ca^2+^ concentration during a voltage clamp depolarization [77, 78] (Fig. 6a, b), or during an action potential or train of action potentials (Fig. 6c) [79] using a calcium-sensitive indicator dye and appropriate optical apparatus [77, 80–82]. However, the measured myoplasmic free Ca^2+^ transient represents only a small fraction of the total Ca^2+^ released during the fiber depolarization. A much larger fraction of the released Ca^2+^ is bound to endogenous myoplasmic Ca^2+^ binding sites (troponin C, parvalbumin, SR Ca^2+^ pump) or transported back to the SR. Taking the Ca^2+^ binding properties of these binding sites and transport into consideration, the Ca^2+^ release flux (rate of Ca^2+^ release) can be calculated [78]. An important first result of such calculations was the conclusion that Ca^2+^ release is *not* maintained during a step depolarization or during a train of action potentials, but instead declines during a 20–50-ms step depolarization (Fig. 6a, middle records) [78] or during a 100-Hz train of actin potentials (Fig. 6c, lower records) [79]. A slower phase of decline of release during longer duration voltage clamp depolarizations also was observed and was attributed to Ca^2+^ depletion from the SR [83, 84], but the faster developing decline of Ca^2+^ release during a voltage clamp pulse or train of APs appears to reflect inactivation of SR Ca^2+^ release. Another important feature of the inactivation is its recovery, as demonstrated using a double-pulse protocol [83]. Here a first conditioning pulse of fixed amplitude and duration is followed by a second pulse of the same amplitude and duration, with a variable time interval between the two pulses (Fig. 6b). For test pulses applied shortly after the conditioning pulse, the time course of the Ca^2+^ release completely lacked the early peak (Fig. 6b). Importantly, the inactivation of Ca^2+^ release during a 20–50-ms pulse does not appear to be due to modification of the voltage sensor since charge movement is not modified during these pulses, as judged by the criteria *Q*on = *Q*off, and *Q* kinetics are not modified after an inactivating prepulse [84].

### Pre-activating and activating components of charge movement for SR Ca^2+^ release

**Fig. 7:**
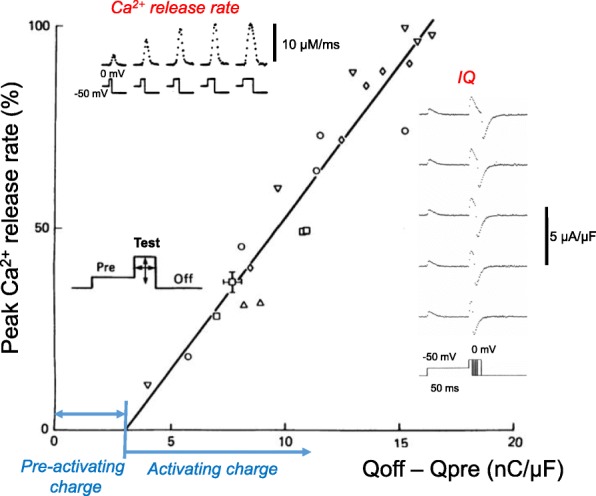
The peak rate of Ca^2+^ release evoked by various depolarizing test pulses is linearly related to the amount of “activation” intramembrane charge moved by the same pulse. Each test pulse was immediately preceded by a depolarizing prepulse (see pulse schematic in lower left inset), which by itself did not activate Ca^2+^ release. Many of the test pulses were too short to establish the ionic current baseline for calculating charge moved during the test pulse, so *Q*on could not be measured for short test pulse durations due to uncertainty regarding the level of ionic current remaining during the test pulse. Charge moved by the test pulse was consequently determined as *Q*off–*Q* pre. The test pulse charge represents an upper estimate of the activating charge moved during the test pulse; the *x* intercept on the graph is interpreted as representing the amount of pre-activating (i.e., precursor) charge still present in the test pulse (i.e., pre-activating charge *not* moved by the prepulse). The lower right inset gives the charge movement current records obtained using various duration test pulses to 0 mV, each immediately following the prepulse to − 50 mV. The upper left inset gives the Ca^2+^ release calculated from the Ca^2+^ transient recorded simultaneously with the charge movement records shown in the lower right inset for each of the same test pulses. Reproduced, with modification from ref. [50]

In theory, “pre-activating” (also termed “sub-threshold” or “threshold”) charge movement would be generated by charge-generating molecular transitions that precede the actual SR Ca^2+^ channel opening event in the signaling pathway from charge movement to RyR1 activation. In contrast, the “activating” charge would coincide with and determine the actual opening of the SR Ca^2+^ release channel. As described above, the pulse duration needed to produce a microscopically just-detectable fiber movement for various voltages was found to be the pulse duration that produced the same constant (= “threshold”) amount of charge moved at each voltage, including during the stepped-on pulse pattern (Fig. 7, inset voltage protocol) [50]. This observation implicates the threshold (or pre-activating) charge as being a precursor that moves before the charge component that actually causes Ca^2+^ release and the subsequent contractile activation. Using Ca^2+^-sensitive dyes and the Ca^2+^ release calculation described in the preceding section, together with the stepped-on pulse protocol (e.g., Fig. 7, lower left inset), it became possible to relate the activating component of charge movement to the rate of SR Ca^2+^ release, the process directly downstream of voltage activated charge movement, and thus directly controlled by the voltage sensor [50].

### Peak SR Ca^2+^ release due to a pulse is proportional to the amount of activating charge moved by the pulse

To relate Ca^2+^ release to charge movement, charge movement was determined for a range of test pulse amplitudes and durations, with each test pulse immediately preceded by the same prepulse, which moved a substantial amount of the pre-activating charge, but did not activate detectible Ca^2+^ release (Fig. 7, right inset). Using these data, it was possible to systematically separate the charge movement that was predominantly precursor (“pre-activating”) for producing SR Ca^2+^ release (and was moved in the prepulse) from charge that was predominantly activating for Ca^2+^ release (and was moved during the test pulse) (Fig. 7, inset right). Ca^2+^ transients were simultaneously measured for the same pulses, and the Ca^2+^ release time course was calculated for each test pulse (Fig. 7, inset top left). Over a wide range of test pulse amplitudes and durations (Fig. 7, voltage protocol), the peak rate of Ca^2+^ release during the test pulse was found to increase linearly with the charge that was moved by the test pulse (Fig. 7). The linear relationship had a small positive charge value for the extrapolation to zero peak rate of release (*x* intercept), indicating a small amount of pre-activating charge that was *not* moved during the subthreshold prepulse, but was instead moved during each test pulse, presumably the initial charge moved during the test pulse. These results demonstrated a close relationship between the extent of activation of SR Ca^2+^ release by a pulse and the amount of activating charge that moved during the same pulse [50].

### Minimal model for voltage sensor control of a coupled RyR1 Ca^2+^ release channel

Figure 5 presents a cartoon of the functional states of the voltage sensor/L-type Ca^2+^ channel in the TT membrane (top) and the RyR1 Ca^2+^ release channel in the SR membrane (bottom) for a hypothetical minimal (two-state) model [85] for regulation of RyR1 by its directly coupled TT voltage sensor(s). In this highly simplified gating scheme, fiber depolarization (top, left to right) causes the mobile voltage sensor charges within the TT membrane voltage sensor protein (Cav1.1), which are constrained to remain within the TT membrane, to respond by generating intra-membrane movement (detected by charge movement measurements [86]). In the minimal scheme of Fig. 5, the voltage sensor charge movement obligatorily induces the opening of the RyR1 Ca^2+^ channel directly coupled to the voltage sensor(s) (bottom; left to right), resulting in a release of Ca^2+^ from the SR, as detected by monitoring the total increase in cytoplasmic and transported Ca^2+^. In this minimal model, there are only two states of the voltage sensor and its directly coupled RyR1 unit: voltage sensor resting/RyR1 closed (left) or voltage sensor active/RyR1 open (right). In addition, in this minimal scheme, the L-type Ca^2+^ channel in the TT membrane is also open when the voltage sensor is active [8].

The minimal model (Fig. 5) already raises several basic issues regarding the TT voltage-dependent gating of RyR1. First, intramembrane charges (transmembrane positively charged S4 alpha helices in the voltage sensor, the Cav1.1) move outward during TT depolarization (i.e., away from the RyR). However, the resulting molecular rearrangements in the voltage sensor accompanying or following charge movement could bring other domains of the Cav1.1 voltage sensor (alpha 1) subunit, or beta subunit either toward or away from the RyR1, so at present we do not know whether the voltage-sensitive step constitutes the removal of an inhibition to RyR1 opening, or the application of a positive factor for voltage-dependent RyR1 activation. This basic issue awaits high-resolution molecular structure-function studies of the Cav1.1/RyR1 interaction in various functional states. Second, the gating scheme in Fig. 5 includes a major simplification. If each of the two ellipses shown in Fig. 5 represents one of the 4 Cav1.1 α1 subunits coupled to a single RyR1 molecule, then each Cav1.1 (i.e., each ellipse) should have four S4 segment charged transmembrane α helices, one in each of the four transmembrane domains of each Cav1.1, rather than the single charged helix shown for each Cav1.1 in the simplified cartoon in Fig. 5.

A clear shortcoming of the minimal scheme in Fig. 5 is that all of the voltage sensor charge movement is directly involved in the closed to open transition of the RyR1 to which it is coupled (i.e., all of the charge moved is “activating” charge movement, as defined above). This property clearly does *not* agree with the experimental characterization of the control system in muscle fibers. An appreciable fraction of the total charge movement that is recorded from a fiber is pre-activating charge, which moves during depolarization, but prior to the charge for the actual activation step in the control mechanism for Ca^2+^ release (above) [50], yet is an essential prerequisite for depolarization-activated SR Ca^2+^ release. In the following sections, we will examine two functional studies that consider more complicated models for voltage sensor/RyR1 interaction(s), as well as other changes occurring in the individual components, leading to the need for increased complexity and refinement of the minimal gating scheme in Fig. 5.

### Model for RyR1 Ca^2+^ release activation requiring simultaneous activation of four identical but independent voltage sensors

**Fig. 8:**
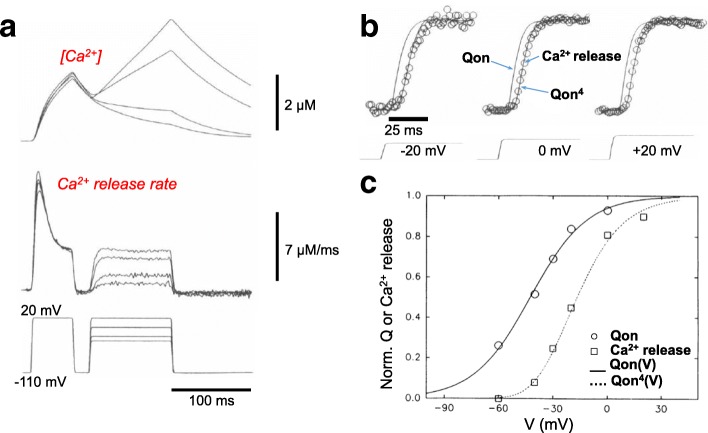
Empirical evidence for a model for activation of each RyR1 in which four identical and independent voltage sensors must all be in the active configuration for the SR Ca^2+^ release channel to open after pre-inactivation of the RyR1 channel. **a** Ca^2+^ transients (top) and calculated rate of SR Ca^2+^ release (middle) for the pulse protocol at the bottom. Pulses to various test pulse amplitudes were each preceded by the same inactivating prepulse, followed by brief return to the holding potential to reset the voltage sensors and close the RyR1 channel and then the various depolarizations (bottom). **b** Wave form of Ca^2+^ release during the test pulses closely follows *Q*^4^. **c** Voltage dependence of Ca^2+^ released follows the fourth power of charge moved (dashed curve). From ref. [88], with modification

In a preceding section and Fig. 7, we considered the empirical (linear) relationship between the peak rate of Ca^2+^ release produced by a given pulse and the activating charge moved in the same pulse [50]. In order to next relate the time course of Ca^2+^ release activation during TT depolarization to the time course of voltage sensor charge movement, it is necessary to consider inactivation of the SR Ca^2+^ release channel, which occurs during fiber depolarization (Fig. 6) [83, 87]. The inactivation process obscures the time course of Ca^2+^ release channel activation, reducing Ca^2+^ release to a fraction of the peak value reached earlier during the pulse (Fig. 6a, b) [87]. One approach eliminated the effects of inactivation during a pulse by pre-inactivating the fibers (Figs. 6b and 8a). Here a large “inactivating” prepulse, sufficient to produce maximal inactivation of RyR1 Ca^2+^ release, was immediately followed by a brief repolarization (to return all charge to the resting state and to turn off the non-inactivating Ca^2+^ release) and then by a test depolarization during which activation of both the “non-inactivating” component of Ca^2+^ release and the charge movement (which does not inactivate in a few hundred ms time window: see above) were monitored (Fig. 8a, b) [83, 88]. Using this approach it was found that both the voltage dependence (Fig. 8c) and time course (Fig. 8b) of the non-inactivating Ca^2+^ release very closely agreed with the voltage dependence and time course of (*Q*/*Q*max)^4^ [88], where *Q*max is the maximum charge, moved during a large depolarization This remarkable finding is consistent with a reaction scheme in which the voltage dependence and kinetics of the non-inactivating component of SR Ca^2+^ release rate is controlled by four identical and independent voltage sensors [88]. Each RyR1 channel would be controlled by four identical voltage sensors and is open *when and only when* all four voltage sensors are in the active conformation, giving rise to release being proportional to the fourth power of *Q*/*Q*max.

If one “voltage sensor” is one Cav1.1 molecule, the voltage sensors could be independent (i.e., in different molecules), and the RyR1 could require all four Cav1.1s to be active in order for RyR1 opening. Indeed, early freeze fracture studies using electron microscopy (EM) revealed that Cav1.1 forms four ordered clusters (tetrads) and the space between tetrads suggested an overall 1:2 ratio of tetrads to Ca^2+^ release channels [89], corresponding to the observation that only half of the RyR1 homotetramers in the triad junction were coupled to Cav1.1 tetrads. However, each Cav1.1 molecule contains four positively charged S4 transmembrane helices, as described below. One possibility is that in the Cav1.1-RyR1 complex in a functioning muscle fiber, interaction between the Cav1.1 and RyR1 gives rise to the condition that only one of the four S4 helices in each Cav1.1 is physically able to move, resulting in a single mobile charged group per Cav1.1, with four independent voltage sensors (one in each Cav1.1 of the tetrad) per coupled RyR1 homo tetramer Ca^2+^ release channel.

### Allosteric mechanisms for RyR channel control by TT Cav1.1 voltage sensor

**Fig. 9:**
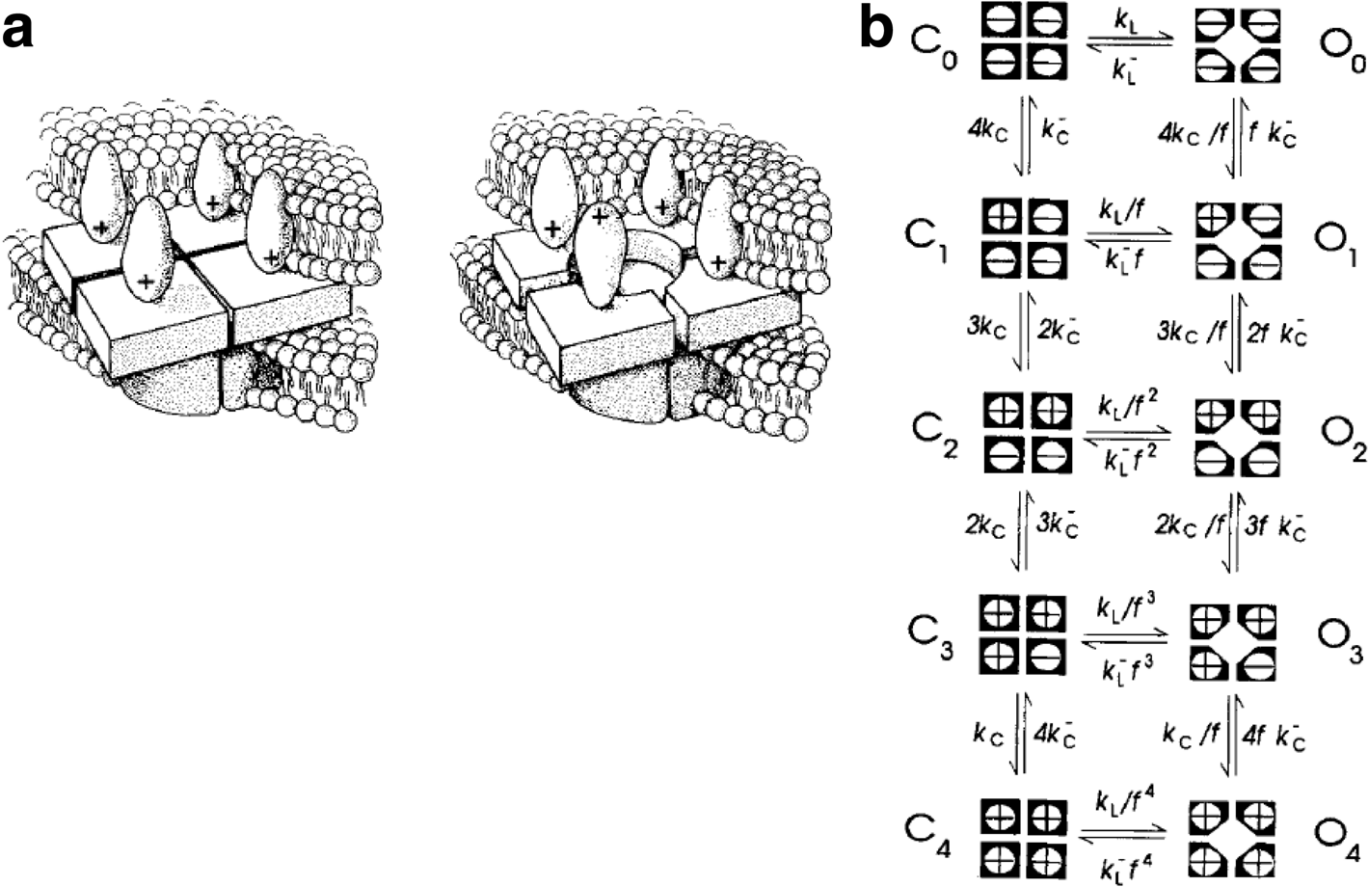
Allosteric model for gating of each coupled RyR1 by four different voltage sensors. **a** Cartoon of a single RyR1 homo-tetramer controlled by four independent voltage sensors, with all four voltage sensors in the inactive configuration (left) or with one voltage sensor in the activated configuration (right). **b** Kinetic reaction scheme for the RyR1 Ca^2+^ channel gating process. Channel opening becomes more likely as more voltage sensors become active, which is the basis for the allosteric effect. However, the channel can open with any number of voltage sensors active. The subscript *i* on C or O is the number of voltage sensors active. Forward rate constant *k* for C*i* → O*i* increases with *i*; the backward rate constant *k* for O*i* → C*i* decreases with *i*. Note that *f* < 1. Elimination of states O_0_ to O_3_ from this scheme reduces it to the model proposed in Fig. 8. From ref. [92], with modification

In the preceding model, the Ca^2+^ release channel opens when and only when a certain enabling configuration of the voltage sensors was achieved. An allosteric model [90, 91] for gating of each RyR1 homo-tetramer by four independent DHPRs provides an alternative approach (Fig. 9a, b). In the allosteric system, each RyR1 channel can open when any number of its coupled voltage sensors is active [92]. However, RyR1 opening becomes increasingly likely as more voltage sensors are active (Fig. 9b). In this model, each RyR1 channel has two states, closed (C) and open (O). Lateral transitions represent opening or closing transitions of the RyR1. Each voltage sensor has two states, inactive or active (− or +, respectively in cartoon). Vertical transitions represent changes in the activation (+) or deactivation (−) status of each of the four voltage sensors, as indicated by the four circles with + or − representing the four voltage sensors controlling each RyR1 [92]. It should be noted that removing the open states O_0_ through O_3_, which are open and have less than four active voltage sensors, and the transitions to and from each of these states removes the possibility of opening without movement of all four voltage sensors, and thereby reduces the allosteric model to the four independent voltage sensor model. Thus, the allosteric model includes the four voltage sensor models, which already fit the data for non-inactivating release very closely [88], as a subset of possibilities. In order to justify the added transitions of the allosteric model, additional experimental data are required and were introduced [92]. Also note that each “voltage sensor” considered here, as in the four independent voltage sensor models above, is an entire Cav1.1, containing a charged transmembrane “S4” helix in each of its four transmembrane domains.

## Molecular components and mechanisms in ECC

### Cav1.1: the TT voltage sensor

**Fig. 10:**
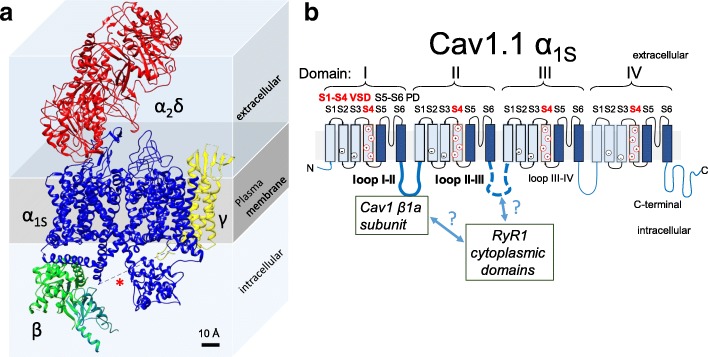
Architecture and membrane topology of the Cav1.1. **a** Side view of a cryo-EM reconstruction of the Cav1.1 and its subunits at 3.6 Å (Protein Data Bank (PDB) 5GJW) [100]. The Cav1.1 α1 subunit is highlighted in blue. Auxiliary β-, α2δ-, and γ-subunits are colored in green, red, and yellow, respectively. This model was created in Chimera [174]. The asterisk symbol indicates the missing II–III loop sequence in the cryo-EM structure. **b** Membrane topology of the Cav1.1 α1 subunits. The α1 subunit is composed by an interconnect array of four homologous (but not identical) domains, each domain consisting of six transmembrane domains, S1–S6. S1–S4 from each domain form a voltage sensor domain, whereas S5 and S6 from all four domains form the pore domain. Intracellular loops connect the domains; the loops II–III and I–II are important for ECC, as indicated

**Fig. 11:**
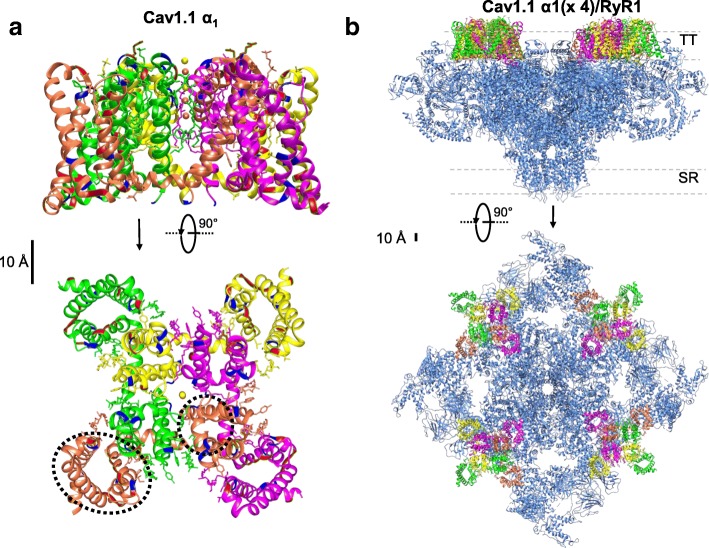
Structural details of the Cav1.1 α1 subunit and the RyR1. **a** Shown are cryo-EM reconstructions of the Cav1.1 a subunit, side view (top) and upper view (bottom) (PDB 5GJW) [100]. The auxiliary subunits, the cytoplasmic tails, and loops are not shown. Each domain is color coded, positively charged residues are indicated in red, negatively charged residues are shown in blue. The dashed ellipse indicates the location of S1–S4 from domain I and the black circle shows the corresponding pore domain (S5–S6) from domain I. Note that each voltage sensor domain (S1–S4) is not in close proximity to its corresponding pore domain (S5–S6). **b** Side (top) and upper (bottom) views of cryo-EM reconstruction of the RyR1 (PDB, 5TAL) [175] and four superimposed Cav1.1 α1 subunits (PDB 5GJW) [100], forming a tetrad. This model was created in Chimera [174] using the cryo-EM maps with relative location of the Cav1.1 subunits as suggested by Samsó [118]

Membrane depolarization of the TT system, during an AP or voltage clamp step depolarization, is detected by Cav1.1 channels, the TT voltage sensor. Cav1.1 channels were initially identified using electrophysiological approaches (charge movement and ionic currents) [37, 56, 69, 70, 93]. Cav1.1 channels are principally expressed in the membrane of the TT system of adult skeletal muscle fibers and are members of a diverse family of voltage-dependent Ca^2+^ channels. Molecular details of the voltage-gated Ca^2+^ channels from skeletal muscle were first identified by binding, purification, and reconstitution [94]. Using molecular biology techniques, their amino acid sequences were determined by cDNA cloning and sequencing [43]. Contrasting with their prominent functional status, TT voltage sensor (Cav1.1 channels) was somewhat apart from the saga of structure-function studies for other types of ion channels [95]. The skeletal muscle Ca^2+^ channel complex is a hetero-tetramer, comprised of a main pore-forming α1 subunit, complexed with β, α2δ, and γ subunits [38, 39, 96] (Fig. 10a) and other ligands (i.e., STAC3) [97]. The Cav1.1 α1 subunit consists of a single polypeptide chain, with four highly homologs but non-identical intramembrane domains (I–IV), each containing six transmembrane (TM) alpha helical segments (S1–S6), shown in cartoon representation in Fig. 10b [43, 96], as well as amino and carboxyl terminals. While the organization of the TM domains of the Cav1.1 α1 subunit has a strong pseudo fourfold symmetry in the plane of the TT membrane, the intracellular structure Cav1.1, including the single β subunit is highly asymmetrical, which could have important implications for Cav1.1-RyR1 coupling. Segments S1–S4 of each transmembrane domain of the α1 subunit form a voltage-sensing domain (VSD) [44, 98], whereas segments S5 and S6 from all four intramembrane domains contribute to the Ca^2+^-conductive pore (Fig. 10b and ribbon diagram in Fig. 11a [98]). The fourth TM helical segment (S4) contains a number of positively charged amino acids (Arg and Lys), separated by two hydrophobic residues (Fig. 10b) [43, 98]. During changes in TT membrane potential, the S4 segments are believed to rearrange, moving outward across the plane of the TT membrane, in response to membrane depolarization, establishing the determinants for voltage sensitivity [9, 99]. Historically, there was a considerable time gap between recognition of the biological importance of Ca^2+^ channels and their structural examination [95], which is changing with the molecular interpretation of their function. Recent cryo-EM studies at a resolution of 3.6 Å [100] revealed more details about the molecular architecture of the Cav1.1 channel of skeletal muscle with its complete set of auxiliary subunits (Fig. 10a). The central α1-subunit of CaV1.1 has a core structure and is associated with an extracellular α2δ-subunit, an intracellular β-subunit, and a 4-TM γ-subunit (Fig. 10a).

### RyR1, the SR Ca^2+^ release channel in skeletal muscle

RyR1-dependent SR Ca^2+^ release via the Ca^2+^ release channel RyR1 initiates muscle contraction. The RyR1 is a colossal protein of approximately 2.3 MDa assembly of four identical subunits [101, 102]. Each subunit contains an intramembrane region, located within the C-terminal region and representing ca. 20% of the total protein, plus a cytoplasmic region that represents 80% of the total protein, and is known as the foot region (Fig. 11b, blue structure; note that the spatial scale is about five times compressed to Fig. 11a, so Cav1.1 appears much smaller in Fig. 11b than a); [103–107]. The cytoplasmic region of the RyR1 channel (280 Å × 280 Å × 120 Å) is continuous with the transmembrane region (120 Å × 120 Å × 60 Å; Fig. 11b) [41]. The RyR1 SR transmembrane region forms the Ca^2+^ release channel [106–109].

RyR1s are arranged in a regular array within the terminal cisternae of the junctional SR [89, 110, 111]. Similarly, Cav1.1 channels are clustered in groups of four (or tetrads) in the TT membrane that is adjacent to the junctional SR [89, 112], with a Cav1.1 tetrad facing every other RyR1 in the junctional SR RyR1 array (Fig. 11b). For symmetry and simplicity, each Cav1.1 molecule composing a tetrad is believed to be oriented in the same coordinated position relative to the subunits of its apposed RyR1homo tetramer (see Fig. 11b) [89, 112]. Since these interfaces take place at alternate RyR1s, half of the RyR1s are “uncoupled” with Cav1.1s, and half are coupled to Cav1.1s [113]. The location of RyR1 (coupled and uncoupled) determines the organization of the Cav1.1 channels in the juxtaposed TT membrane, creating a “checkerboard” array of coupled and uncoupled RyR1s that produces the Cav1.1 lattice organization [114]. Depolarization-induced activation of RyR1 is believed to be mediated via direct or indirect interactions with TT voltage sensors [8, 115–117]. However, despite extensive biophysical and ultrastructural studies, the molecular basis for TT voltage sensor function and the chemical mechanisms that support TT voltage regulated RyR1 SR Ca^2+^ release, as well as the orientation of the Cav1.1 tetrads relative to the RyR1 have remained unclear. In Fig. 11b, the location of the tetrads is based on reference [118], whereas the relative orientation is arbitrary.

The RyR1 components, including SR luminal segments, transmembrane domains, and large cytosolic domains, and their interaction with the Cav1.1 channels, SR luminal proteins, and accessory proteins, metabolites, and ions, as well as post-translational modifications, allow the RyR1s to be fine-tuned by numerous mechanisms [71, 107, 109, 119–128]. However, the TT voltage sensor (CaV1.1) is believed to be the “ligand” that uniquely enables RyR1 opening in functioning skeletal muscle fibers.

### The TT voltage sensor is the master regulator of RyR1

Despite the large number of modulatory interactions that influence RyR1 activation, it is difficult to overemphasize the importance of the TT voltage sensor, and/or some component(s) directly coupled to it, in the physiological regulation of RyR1 in skeletal muscle. Indeed, the voltage sensor can be considered as the master ligand for controlling SR Ca^2+^ release in normal mature muscle fibers. First, no other component, except possibly Ca^2+^ influx into the TT/SR gap, would be anticipated to change drastically during the few millisecond action potential which raises cytoplasmic Ca^2+^ sufficiently to cause a twitch contraction. However, Ca^2+^ influx is not needed for skeletal muscle activation [73], which is maintained for depolarizations well beyond the Ca^2+^ reversal potential, where Ca^2+^ influx is greatly suppressed or eliminated [74]. Second, the frequency of occurrence of Ca^2+^ sparks, elementary intracellular Ca^2+^ signals [129, 130], in frog skeletal muscle fibers can be used as a measure of activation of “microscopic” Ca^2+^ release events, which combine at high frequencies during fiber depolarization to produce the “macroscopic” Ca^2+^ transient observed during depolarization [131, 132]. These events are extremely infrequent in resting fibers, but increase tremendously in frequency during depolarization, so much so that spark frequency can only be monitored experimentally during large depolarizations by using depolarized fibers, and restoring only a small fraction of the release units by brief repriming repolarizations [130, 133–135]. These types of experiments indicate that in functioning muscle fibers, the RyR1 SR Ca^2+^ release channels are essentially fully off when the voltage sensors are in the resting condition, but turn on strongly and rapidly during the AP or voltage clamp depolarization that activates the voltage sensors [132, 135, 136]. The simplest hypothesis is that under normal conditions in mature functioning muscle fibers, RyR1s coupled to TT voltage sensors are locked in the off configuration due to an inhibitory influence of the voltage sensor in its resting configuration [137]. Movement of the voltage sensor into the active configuration during depolarization removes this “lock” on RyR1 opening, and the RyR1 channels open and, more slowly, inactivate (Fig. 6a) [78]. When the voltage sensor returns to the resting configuration at the end of the depolarization, the RyR1 is relocked. In this scenario, all the other ligands that modulate RyR1 channel activity in isolated membrane or protein preps may or may not also similarly modulate the RyR1 in a functioning fiber, but the TT voltage sensor serves as master regulator determining whether or not the channel can open at all.

### Dysgenic muscle and restoration of skeletal vs. cardiac ECC

Advances in biochemistry, molecular biology, and pharmacology allowed the identification of the molecular components that are essential for ECC. One crucial discovery was the characterization of a naturally occurring “knock out” of the Cav1.1 α1 subunit (“dysgenic” mouse; [138]). This model demonstrated that myotubes derived from the dysgenic mice lacked ECC and intramembrane charge movement; the expression of α1s subunit (skeletal muscle isoform) in these cells restored “skeletal” type of ECC, which is independent of Ca^2+^ influx [139]. The expression of the cardiac isoform (α1c subunit) of the Cav1 channels did not restore skeletal ECC [140]. The inability of the cardiac isoform [140] and of other Ca^2+^ channel subtypes [141, 142] to rescue the ECC allowed the investigation of essential elements for skeletal muscle ECC via chimeric channels.

### Components of Cav1.1 that are needed for activating RyR1

Chimeric channels made with α1 subunits of Cav1.1 and Cav1.2 demonstrated that a region in the intracellular loop between the second and third domains (II–III loop), specifically, the region spanning residues 720–764/5, was important for this function (Fig. 10b) [143, 144]. Interestingly, in the cryo-EM structure of Cav1.1 the structure of the II–III loop is undefined (dashed line in Fig. 10a, [100]), whereas the I–II and III–IV/C-terminal regions are defined and appear in Fig. 10a. While the identification of the Cav1.1 regions that are critical for ECC has been more active, perhaps due to the smaller size of the Cav1.1 channel, the identification of binding domains in the RyR1 for the II–III loop has been less fruitful. Only a few reports, where deletions of large segments of the RyR1 successfully altered ECC, concluded that several regions of the RyR1 are involved in the interaction with Cav1.1 [145–147]. Recent approaches are revisiting the role of the loop II–III and its association with the adaptor protein STAC3 on ECC [148]. These new results support the notion that the II–III linker plays a role in ECC.

The Cav1.1 I–II loop is the site for interaction with the β1a subunit (Fig. 10b) [98]. The β1a subunit is important for several aspects of ECC. The β1a subunit is needed for the functional expression of Cav1.1 α1 subunit [149] and is crucial for enhancement of Cav1.1 α1 triad expression [150], assembly of Cav1.1 α1 in tetrads [151, 152], and elicitation of Cav1.1 α1 charge movement [153]. Skeletal-type ECC is reduced in muscle cells lacking the expression of β1a [150] and is rescued by expression of β1a [154]. The use of chimeric constructs of β1a [155] with other β subunits, as well as the use of synthetic peptides [156], allowed the identification of the C-terminal region of β1a as an important domain for possible interaction with RyR1 during TT voltage-dependent SR Ca^2+^ release.

Thus, several sites in the Cav1.1 α1 subunit, in addition to the II–III loop, contribute to the overall Cav1.1/RyR interaction. These include the loop I–II and β-subunit [157, 158], and more indirectly, the III–IV loop [159] and the C-terminal domain of the Cav1.1 α1 [141] (Fig. 10)b.

Another important advance in the characterization of the molecular players of the ECC and their interactions was the generation of a RyR1-knockout mouse (the dyspedic mouse [160]), which allowed for the expression of various RyR constructs and different Cav1.1 α1/RyR1 combinations [161, 162]. These approaches identified that the skeletal Cav1.1 α1-subunit and RyR1 are essential for the skeletal muscle function. Skeletal ECC was not experimentally evident if Cav1.1/RyR1 were not in the membrane, or forming tetrads.

### Discrete S4 voltage sensor (i.e., S4, I–S4, IV) models

In voltage-gated sodium channels, a channel structurally and evolutionary similar to Cav1.1 [39], studies using mutagenesis and voltage-clamp fluorometry revealed that the four VSDs, each linked to a partial pore-forming region, may be differentially and allosterically coupled to the pore opening to various degrees of involvement in the control of voltage dependence and gating [163]. VSDs I–III activate in parallel and sufficiently rapidly to modulate Na^+^ channel opening, whereas VSD IV activates more slowly and initiates fast inactivation [163]. In Cav1.2, a voltage-gated Ca^2+^ channel expressed in cardiac cells, site-directed fluorophore labeling, and voltage clamp fluorometry of individual VSDs showed differential function for each domain; VSDs II and III exhibited voltage-dependent and kinetic characteristics compatible with channel activation [164]. However, the cardiac isoform (α1c subunit) is unable to support RyR1 activation in myotubes lacking α1s [140, 144].

**Fig. 12:**
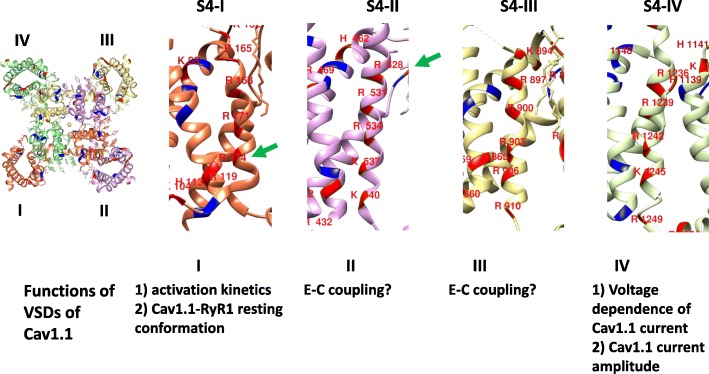
Voltage sensing domains of Cav1.1. From left to right, S4-I, S4-II, S4-III, and S4-IV. The positively charged residues of the S4 segments are labeled in red for each domain. Negatively charged amino acids are shown in blue. The depicted transmembrane helices are from the structure of the cryo-EM of Cav1.1 (PDB 5GJW) [100]. These models were created using Chimera [174]. Refs. for VDSI [165, 170], VDSII [165, 171], VSD III [165, 167], and VSD IV [165–167]

Similarly, the voltage dependence and timing of Ca^2+^ entry via Cav1.1, as well as the voltage dependence and timing of TT voltage-dependent RyR1 Ca^2+^ release, are expected to be functions of the α1-subunit of Cav1.1, which also contains four highly similar but non-identical VSDs, I–IV [43, 96, 98]. Evidence for a differential role of each VSD in Cav1.1 channel operation using chimeric studies (interchange of VD SI region, Cav1.1↔Cav1.2) and alternative splicing of VSD IV of Cav1.1 suggests that VSDs I and IV control the activation kinetics and voltage dependence, respectively [165–167]. Because VSD I and VSD IV appear to be linked to the slow activating Cav1.1 ionic current, it was hypothesized that VSDs I and IV do not contribute to the more rapid Cav1.1-dependent SR Ca^2+^ release [168, 169] (see Fig. 12). In support of this hypothesis, a functional study of a mutation causing malignant hyperthermia susceptibility (R174W) in S4 of VSD I of Cav1.1 revealed that this mutation reduces Cav1.1 ionic current, but does not affect Cav1.1-dependent SR Ca^2+^ release [170] (Fig. 12). Wu and colleagues [171] used a mouse model for hypokalemic periodic paralysis with a targeted Cav1.1 R528H mutation in S4 of VSD II. Muscle fibers from the Cav1.1 R528H homozygous mouse exhibited impaired depolarization-induced Ca^2+^ release, suggesting that VSD II could participate in Cav1.1-dependent SR Ca^2+^ release [171] (Fig. 12). These results represent compelling but still indirect evidence of the role of each VSD in Cav1.1. Currently, the contribution of the individual VSDs to the voltage dependence of Cav1.1 pore opening and activation of RyR1 Ca^2+^ release is unknown. Note that the Cav1.1 R528H mutation also introduces a “gating pore” or “omega” current, which is normally not present in the wild-type channel and is responsible for the anomalous depolarization seen in hypokalemic periodic paralysis [171]. Details regarding the gating pore current have been reviewed in [172, 173].

## Future perspectives

How does the propagated electrical impulse spreading along the TT system produce Ca^2+^ release? The Schneider and Chandler hypothesis that the excitatory signal passes from the TT to the SR membrane by way of charges moving in the TT membrane connecting with the junctional feet of the SR Ca^2+^ release channel initiated the path to answer this question. Yet, it is still unknown how this movement of charge, originating in Cav1.1, transfers a signal across to the RyR1 (feet) to trigger Ca^2+^ release. This question is particularly fascinating because of the as yet unknown molecular structure-function relationship between these components in two different membrane systems, the TT (Cav1.1 voltage sensors) and the SR (RyR1 Ca^2+^ release channels). Cryo-EM has revealed amazing details of the structure of the Cav1.1 (in a closed configuration) and of the RyR1 (in closed and ligand-induced open conformations). The next generation of high-resolution cryo-EM, together with electrophysiological assays using chimeric constructs or site-directed mutagenesis, may provide a more comprehensive molecular picture of the interaction between Cav1.1 and RyR1 in their respective membranes.

## Conclusions

Electrophysiological studies and more recently, the solution of the structures of the Cav1.1 and the RyR1 at near-atomic level, have provided in depth functional and structural details of the ECC process. However, it is clear that new approaches are needed to continue to explore the intricacies of ECC. Some of the many remaining unanswered questions regarding ECC include the following: Are all four VSDs (I–IV) needed to activate the RyR1 Ca^2+^channel, or is only a subset of charges involved? If so, which VSDs are coupled to Cav1.1 pore opening? Which VSDs contribute the voltage sensor element(s) for electromechanical coupling between the Cav1.1 and RyR1 Ca^2+^ release? Which residues are moved during Cav1.1 channel activation? Which residues are moved for RyR1 Ca^2+^ release channel activation? How far do they move within the membrane electric field? And what are the molecular determinants that mediate the electromechanical coupling between the VSD and RyR1 Ca^2+^ release? These are some of the interesting questions for current and future investigation.

## Abbreviations

AP: Action potential
Ca_v_1.1: Voltage-dependent Ca^2+^ channel skeletal muscle isoform 1.1
Cryo-EM: Cryogenic electron microscopy
DHPR: Dihydropyridine receptor
ECC: Excitation-contraction coupling
EGTA: Ethylene glycol-bis(2-aminoethylether)-N, N, N′, N′-tetraacetic acid
*IQ*(*t*): Non-linear capacitive currents
*k*: A measure of steepness in the Boltzmann function
*Q*(*t*): Charge movement
*Q*max: Maximum charge per unit of linear capacitance
*Q*off: Charge moved during the repolarization phase of the test pulse
*Q*on: Charge moved during the beginning of a test pulse
RyR1: Ca^2+^ release channel type 1
SR: Sarcoplasmic reticulum
TT: Transverse tubules
Vh: The mid-point in axis voltage for ½ of *Q*max
VSD: Voltage sensor domain
